# Airway epithelial cell necroptosis contributes to asthma exacerbation in a mouse model of house dust mite-induced allergic inflammation

**DOI:** 10.1038/s41385-021-00415-5

**Published:** 2021-05-27

**Authors:** Nikos Oikonomou, Martjin J. Schuijs, Antonis Chatzigiagkos, Ariadne Androulidaki, Vassilis Aidinis, Hamida Hammad, Bart N. Lambrecht, Manolis Pasparakis

**Affiliations:** 1 Institute for Genetics, Cologne Excellence Cluster on Cellular Stress Responses in Aging-Associated Diseases (CECAD) & Center for Molecular Medicine, University of Cologne, Cologne, Germany; 2 Laboratory of Immunoregulation and Mucosal Immunology, VIB-UGent Center for Inflammation Research, Ghent, Belgium; 3 Biomedical Sciences Research Center ‘Alexander Fleming’, Vari, Greece; 4 Department of Internal Medicine and Pediatrics, Ghent University, Ghent, Belgium; 5 Department of Pulmonary Medicine, Erasmus University Medical Center Rotterdam, Rotterdam, the Netherlands

## Abstract

Regulation of epithelial cell death has emerged as a key mechanism controlling immune homeostasis in barrier surfaces. Necroptosis is a type of regulated necrotic cell death induced by receptor interacting protein kinase 3 (RIPK3) that has been shown to cause inflammatory pathologies in different tissues. The role of regulated cell death and particularly necroptosis in lung homeostasis and disease remains poorly understood. Here we show that mice with Airway Epithelial Cell (AEC)-specific deficiency of Fas-associated with death domain (FADD), an adapter essential for caspase-8 activation, developed exacerbated allergic airway inflammation in a mouse model of asthma induced by sensitization and challenge with house dust mite (HDM) extracts. Genetic inhibition of RIPK1 kinase activity by crossing to mice expressing kinase inactive RIPK1 as well as RIPK3 or MLKL deficiency prevented the development of exaggerated HDM-induced asthma pathology in FADD^AEC-KO^ mice, suggesting that necroptosis of FADD-deficient AECs augmented the allergic immune response. These results reveal a role of AEC necroptosis in amplifying airway allergic inflammation and suggest that necroptosis could contribute to asthma exacerbations caused by respiratory virus infections inducing AEC death.

## Introduction

Asthma is a chronic inflammatory disorder of the airways that is characterized by eosinophilia and lymphocytosis, goblet cell metaplasia, smooth muscle activation and airway hyperreactivity. In most cases of asthma this chronic airway inflammatory response is orchestrated by the secretion of type 2 cytokines like IL-4, IL-5 and IL-13 from activated T_H_2 cells.^1,2^ Accumulating evidence suggests that epithelial cells of the lung play important roles in inducing type 2 immune responses against inhaled allergens such as House Dust Mites (HDM). Acting as barrier, sentinel cells, airway epithelial cells (AECs) recognize allergens with Pattern Recognition Receptors like TLR4 and C-type lectin receptors or protease-activated receptors and respond by releasing cytokines like TSLP, IL-33, IL-1α, GMCSF and chemokines such as CCL2, CCL3 and CCL20, all of which induce a pro-allergic phenotype on Dendritic Cells (DCs).^3^

Besides their contribution to the activation of type 2 innate and adaptive immune responses, AECs are also the main targets of numerous inhaled toxicants and inflammatory mediators that are released by activated T cells, eosinophils or other immune cells that can cause cell death. Shedding of AECs is a typical feature in biopsies from asthma patients^4–6^ and ultrastructural studies have indicated increased frequency of cell death in AECs especially in cases of severe asthma.^7^ The increased occurrence of apoptotic cells in pediatric asthma^8^ and the presence of desquamated epithelial cells known as Creola bodies in the sputum of infants with viral bronchiolitis, which has been correlated with the subsequent development of wheezing and asthma,^9,10^ indicate a link between epithelial cell death and the pathogenicity of asthma. Moreover, persistence of dying AECs due to insufficient clearance exacerbated allergic airway inflammation.^11^

In addition to the structural consequences of epithelial damage, death of AECs might also affect immunological parameters of allergic airway inflammation. A recent study suggested that HDM causes DNA double strand breaks in AECs that correlated with increased presence of cleaved caspase 3^+^ cells and elevated cytokine production.^12^ The propensity of HDM to induce type 2 immune responses correlates with its ability to cause the release of uric acid,^13^ ATP,^14^ IL-1α^15^ and IL-33 from AECs. Notably, these endogenous factors known as alarmins or Danger Associated Molecular Patterns are potent inducers of immune responses that are released from cells undergoing immunogenic types of cell death.^16–18^ Recent research has highlighted the importance of regulated cell death in tissue homeostasis and inflammation.^19,20^ Necroptosis has been recently identified as a new type of regulated necrotic cell death that stimulates potent inflammatory responses.^19,21^ Receptor interacting protein kinase 3 (RIPK3) induces necroptosis by phosphorylating the pseudokinase Mixed lineage kinase like (MLKL), which executes cell death by damaging the plasma membrane.^19,21^ RIPK1 is a key mediator of necroptosis downstream of death receptors or Toll-like receptors (TLR3 and TLR4). RIPK1 exhibits kinase-independent scaffolding functions that inhibit cell death and favor cell survival and NF-κB-dependent inflammatory gene expression.^22,23^ On the other hand, activation of the kinase activity of RIPK1 facilitates the formation of death-inducing protein complexes that trigger cell death by activating apoptosis or necroptosis. Genetic or pharmacological inhibition of caspase-8 or the Fas-Associated with Death Domain (FADD) adapter protein favors the formation of the necrosome, a protein complex facilitating the activation of RIPK3 and subsequent phosphorylation of MLKL for the induction of necroptosis.^19^ Genetic mouse model studies showed that FADD or caspase-8 deficiency caused RIPK3-MLKL-dependent necroptosis in epithelial cells causing inflammation in barrier tissues such as the skin and the intestine, identifying epithelial cell necroptosis as a potent driver of inflammation.^24–27^ Although apoptosis is generally considered non-immunogenic, accumulating evidence suggests that FADD-caspase-8-dependent cell death can also cause inflammation.^19^

The role of RIPK3-MLKL-induced necroptosis and FADD-caspase-8-dependent apoptosis in lung homeostasis, inflammation and disease remains poorly explored. Here we investigated the potential involvement of AEC necroptosis and apoptosis in the development of HDM-induced asthma-like pathology. Our results showed that mice with AEC-specific deficiency in FADD (FADD^AEC-KO^) developed exaggerated allergic airway inflammation in response to HDM sensitization and challenge compared to their *Fadd*^fl/fl^ littermates. Inhibition of RIPK1 kinase activity as well as RIPK3 deficiency both prevented the excessive activation of HDM-induced allergic inflammation in the lungs of FADD^AEC-KO^ mice, suggesting that AEC necroptosis greatly contributes to the amplification of the immune response.

## Results

### AEC-specific ablation of FADD exaggerates HDM-induced airway inflammation

**Fig. 1:**
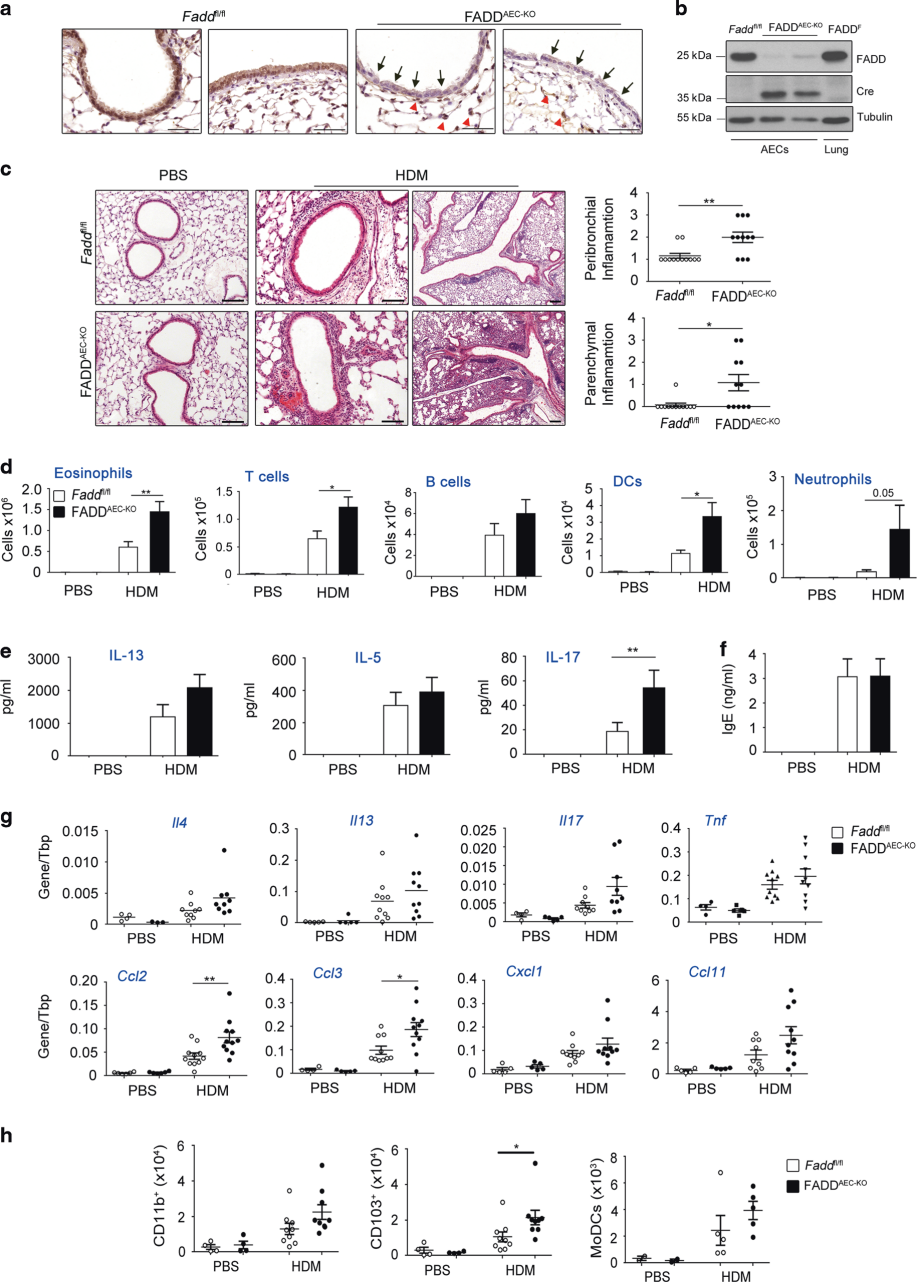
Airway epithelial FADD deficiency results in exacerbated HDM-induced allergic inflammation. **A** Representative images of lung sections from the indicated genotypes immunostained for FADD. Bronchioles (left panel) and bronchi (right panel) are depicted. Arrows indicate AECs and arrowheads indicate parenchymal or immune cells (*n* = 5 per genotype) Scale bar, 50 μΜ. **B** Immunoblot analysis for FADD and Cre expression in lysates of AECs or lung tissue isolated from the indicated genotypes. Tubulin is used as a loading control. **C** Representative images of H&E stained lung sections from *Fadd*^fl/fl^ and FADD^AEC-KO^ mice sensitized and challenged with PBS or HDM and clinical scores of peribronchial and parenchymal inflammation. Scale bars, 100 and 200 μM. **D** Differential cell count analysis with flow cytometry in BALF cells from the indicated genotypes. **E** Levels of cytokines in the supernatants of MLN cells after ex vivo stimulation with HDM. **F** Serum levels of total IgE. **G** qRT-PCR analysis of the indicated cytokines in whole lung mRNA isolated from *Fadd*^fl/fl^ and FADD^AEC-KO^ mice. **H** FACS analysis of DC subsets recruited in MLNs 3 days after HDM exposure. For **C**–**G** data are pooled from two experiments, *n* = 9–11 for HDM, *n* = 4–6 for PBS. For **H** data from two experiments for CD11b and CD103 with *n* = 3–9 and one experiment for MoDCs with *n* = 3–5. Error bars indicate SEM. *P* values reflect the Mann–Whitney U test: *P < 0.05, **P < 0.01, ***P < 0.001.

In order to study the role of epithelial cell death in the development of HDM-induced immunopathology, we generated mice with AEC-specific deletion of FADD (FADD^AEC-KO^) by crossing mice carrying *Fadd* floxed alleles (*Fadd*^fl/fl^)^28^ with *Scgb1a1*-Cre transgenic mice expressing Cre under the control of the rat *Scgb1a1* promoter.^29^ Immunohistochemical analysis of lung sections from FADD^AEC-KO^ mice with anti-FADD antibodies revealed efficient ablation of FADD in bronchial and bronchiolar epithelial cells, while neighboring alveolar epithelial and immune cells expressed the protein (Fig. 1A). Furthermore, immunoblot analysis of primary AEC lysates showed strong reduction of FADD expression in FADD^AEC-KO^ mice (Fig. 1B). FADD^AEC-KO^ mice were born at a Mendelian ratio and did not develop any apparent abnormalities, showing that FADD ablation in AECs did not disrupt normal lung homeostasis.

To study how AEC-specific FADD ablation affected the pathogenesis of allergic airway inflammation we analyzed the response of FADD^AEC-KO^ mice and their *Fadd*^fl/fl^ littermates to a well-established model of asthma induced by instillation of HDM.^15^ To this end, mice were sensitized with intranasal instillation of 1 μg HDM on day 0 followed by challenge with 10 μg HDM on 5 consecutive days starting 1 week after sensitization. Mice were analyzed 3 days after the last HDM instillation. *Fadd*^fl/fl^ mice, as expected, developed all the cardinal features of asthma including increased infiltration of immune cells, T_H_2 cytokine production by Mediastinal Lymph Nodes (MLN) cells and increased titers of IgE antibodies in the serum (Fig. 1D, E, F). In contrast, FADD^AEC-KO^ mice exhibited an exacerbated inflammatory phenotype characterized by severe eosinophilia, lymphocytosis and increased infiltration of CD11c^+^MHCII^high^ DCs and CD11b^+^GR-1^+^ neutrophils (Fig. 1C, D). Toluidine blue staining of lung sections revealed no difference in the recruitment of mast cells in the lungs of FADD^AEC-KO^ compared to *Fadd*^fl/fl^ mice (data not shown). Moreover, in ~40% of FADD^AEC-KO^ mice infiltration of inflammatory cells was not limited around airways and blood vessels but instead expanded into the parenchyma, which in the most severe cases obliterated the alveolar compartment of at least one lung lobe (Fig. 1C, HDM). The cellular infiltrate consisted mainly by eosinophils and contained macrophages that appeared multinucleated bearing the morphological characteristics of giant cells. Further histopathological examination of lung sections revealed the presence of dense cellular aggregates around airways reminiscent of inducible bronchus-associated lymphoid tissue (iBALT). These structures were much more common in the lungs of FADD^AEC-KO^ mice, especially in those that were more severely affected. Quantitative PCR analysis of the expression levels of *Cxcl1*3, a chemokine known to induce iBALT through the recruitment of cells like B cells and Tfh cells, showed that FADD^AEC-KO^ mice produced higher levels of the chemokine when compared to *Fadd*^fl/fl^ mice (Fig. S1A), suggesting that FADD deficiency in AECs can potentiate iBALT formation upon allergen sensitization and challenge. MLN cells from FADD^AEC-KO^ mice produced mildly elevated amounts of IL-13 but similar amounts of IL-5 compared to cells from *Fadd*^fl/fl^ littermates when re-stimulated ex vivo with HDM (Fig. 1E). However, MLN cells from FADD^AEC-KO^ mice secreted increased amounts of IL-17A compared to cells from *Fadd*^fl/fl^ mice, indicating that epithelial cell FADD deficiency results in an amplification of T_H_17 responses (Fig. 1E). Serum IgE levels were equal between the two genotypes (Fig. 1F). Quantitative RT-PCR analysis of mRNA isolated from lung tissue showed mildly increased expression of *Il-4, Il-13, Tnf, Cxcl1, Ccl11* and *Il-17*, as well as statistically significant increase of *Ccl2* and *Ccl3* expression in FADD^AEC-KO^ compared to *Fadd*^fl/fl^ mice (Fig. 1G). Sensitization to HDM depends on the activation of DCs and their migration from the lungs to the draining lymph nodes, where they activate naïve CD4^+^ T cells into T_H_2 effector cells.^30^ Analysis of DC subsets in MLNs 3 days after a single HDM instillation using flow cytometry revealed that the numbers of the different DC subsets were evidently increased in the lymph nodes of FADD^AEC-KO^ when compared to *Fadd*^fl/fl^ mice, with a predominance of CD103^+^ DCs (Fig. 1H). Collectively, these results showed that FADD ablation in AECs exacerbated HDM-induced inflammation, an effect that appears to be the result of the coordinated actions of both adaptive and local innate immune activation.

### RIPK1 kinase activity drives exacerbated HDM-induced airway inflammation in FADD^AEC-KO^ mice

Recent studies highlighted the importance of RIPK1 as a regulator of cell death and inflammation.^31,32^ Whereas RIPK1 kinase activity induces apoptosis and necroptosis, RIPK1 exhibits also kinase-independent scaffolding functions that inhibit apoptosis and necroptosis.^19,33,34^ In order to investigate the role of RIPK1 in AECs we generated RIPK1^AEC-KO^ mice by crossing the *Scgb1a1*-Cre mice to mice bearing loxP-flanked *Ripk1* alleles.^31^ In contrast to *Ripk1*^−/−^ mice, which die perinatally exhibiting a multi-organ inflammatory response that is caused by FADD-caspase-8-mediated apoptosis and RIPK3-MLKL-dependent necroptosis,^22,23,35^ RIPK1^AEC-KO^ mice were born at Mendelian ratio and were healthy and fertile with no apparent pulmonary pathology (data not shown). To assess whether epithelial RIPK1 deficiency affects the pathogenesis of asthma we analyzed the response of RIPK1^AEC-KO^ and *Ripk1*^fl/fl^ littermates to HDM-induced airway inflammation. HDM sensitization and challenge of RIPK1^AEC-KO^ mice induced features of asthma pathology that were indistinguishable from the disease developing in their *Ripk1*^fl/fl^ littermates (Fig. S2A–D), showing that AEC-specific deletion of RIPK1 did not considerably affect HDM-induced allergic airway inflammation.

**Fig. 2:**
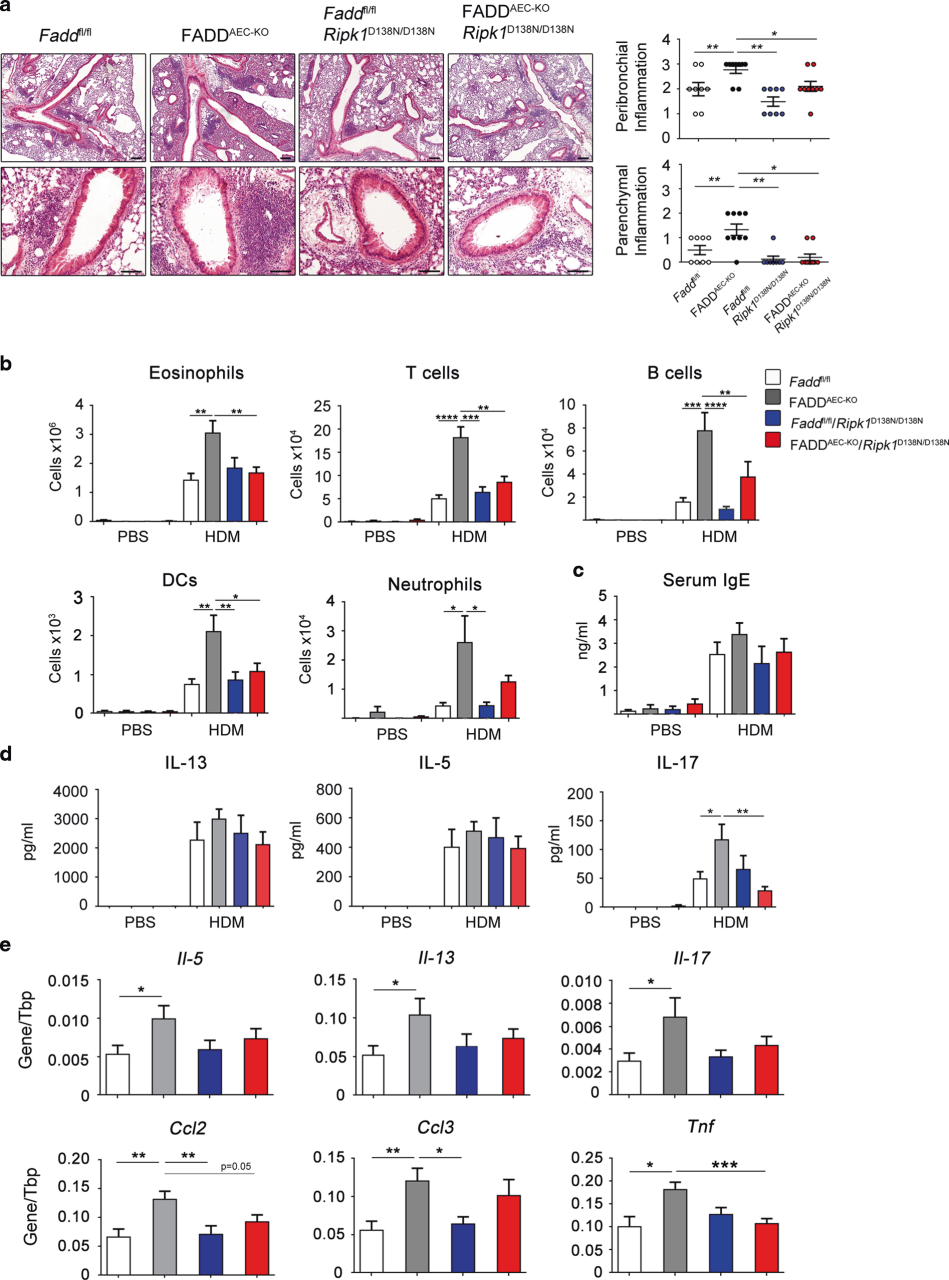
RIPK1 kinase activity drives exacerbated HDM-induced airway inflammation in FADD^AEC-KO^ mice. **A** Representative images of H&E stained lung sections from the indicated genotypes sensitized and challenged with PBS or HDM and clinical scores of peribronchial and parenchymal inflammation. Scale bar, 100 and 200 μΜ. **B** Differential cell count analysis with flow cytometry in BALF cells from the indicated genotypes. **C** Serum levels of total IgE. **D** Levels of cytokines in the supernatants of MLN cells after ex vivo stimulation with HDM. **E** qRT-PCR analysis of the indicated cytokines in whole lung mRNA. Pooled data from two experiments, *n* = 8–11 for HDM, *n* = 4 for PBS. Error bars indicate SEM. *P* values reflect the Mann–Whitney U test: *P < 0.05, **P < 0.01, ***P < 0.001.

We reasoned that AEC-specific FADD deficiency might cause exacerbated airway inflammation in response to HDM by sensitizing AECs to necroptosis, as was previously shown in mice lacking FADD specifically in epidermal keratinocytes or intestinal epithelial cells.^24,25^ RIPK1 kinase activity is required for TNFR1- and TLR3/4-induced necroptosis in cells that lack FADD or caspase-8 or are treated with caspase inhibitors.^19^ To address whether the kinase activity of RIPK1 might be implicated in the development of more severe HDM-induced airway inflammation in FADD^AEC-KO^ mice, we crossed them with *Ripk1*^D138N/D138N^ mice expressing a kinase inactive mutant form of RIPK1.^36^ Assessment of HDM-induced airway inflammation revealed that *Fadd*^fl/fl^ *Ripk1*^*D138N/D138N*^ mice developed similar lung pathology compared to *Fadd*^fl/fl^ animals (Fig. 2A–C), showing that lack of RIPK1 kinase activity did not affect HDM-induced asthma pathology in mice with intact FADD signaling. However, comparison of HDM-induced airway inflammation between FADD^AEC-KO^ and FADD^AEC-KO^ *Ripk1*^D138N/D138N^ mice revealed that lack of RIPK1 kinase activity strongly attenuated the exacerbated HDM-induced pathology observed in FADD^AEC-KO^ mice as judged by histopathological examination of lung sections (Fig. 2A), assessment of immune cell infiltration (Fig. 2B) and measurement of serum IgE levels (Fig. 2C). Moreover, loss of RIPK1 kinase activity significantly suppressed IL-17A production in ex vivo restimulated MLN cells from FADD^AEC-KO^*Ripk1*^D138N/D138N^ mice (Fig. 2D), suggesting a critical role of the kinase activity of RIPK1 in mediating allergen-induced T_H_17 responses when FADD signaling is compromised in AECs. In addition, qRT-PCR analysis of innate and adaptive immune cytokines and chemokines revealed that FADD^AEC-KO^*Ripk1*^D138N/D138N^ mice expressed reduced levels of *Il-5*, *Il-13* and *Il-17A* as well as *Ccl2*, *Ccl3* and *Tnf* in their lungs compared to FADD^AEC-KO^ mice (Fig. 2E). Collectively, these results showed that RIPK1 kinase activity is required for the development of exacerbated HDM-induced airway inflammation in FADD^AEC-KO^ mice, suggesting that RIPK1-mediated necroptosis of FADD-deficient AECs contributes to the more severe pathology.

### RIPK3-MLKL-dependent AEC necroptosis causes exacerbated HDM-induced airway inflammation in FADD^AEC-KO^ mice

**Fig. 3:**
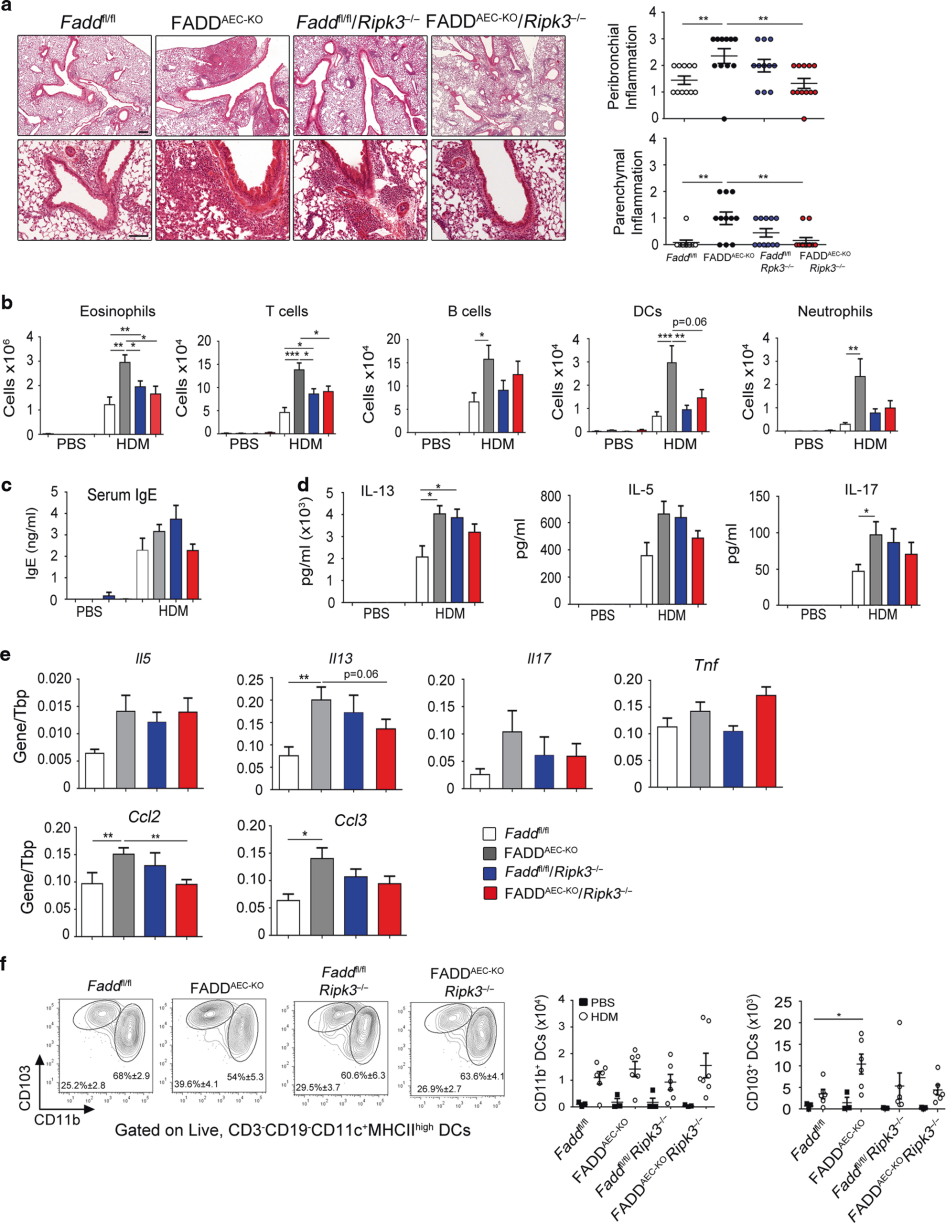
RIPK3-dependent AEC necroptosis causes exacerbated HDM-induced airway inflammation in FADD^AEC-KO^ mice. **A** Representative images of H&E stained lung sections from the indicated genotypes sensitized and challenged with PBS or HDM and clinical scores of peribronchial and parenchymal inflammation. Scale bar, 100 and 200 μΜ. **B** Differential cell count analysis with flow cytometry in BALF cells from the indicated genotypes. **C** Serum levels of total IgE. **D** Levels of cytokines in the supernatants of MLN cells after ex vivo stimulation with HDM. **E** qRT-PCR analysis of the indicated cytokines in whole lung mRNA. **F** FACS plots and absolute numbers of CD11b^+^ and CD103^+^ DC subsets that migrated to MLNs 3 days after HDM instillation. For **A**–**E** pooled data from two experiments, *n* = 10–11 for HDM, *n* = 3 for PBS. For **F** pooled data from two experiments, *n* = 6 for HDM, *n* = 3 for PBS. Error bars indicate SEM. *P* values reflect the Mann–Whitney U test: *P < 0.05, **P < 0.01, ***P < 0.001.

To directly assess the potential contribution of RIPK3-mediated necroptosis of FADD-deficient AECs in exacerbating HDM-induced airway inflammation, we sensitized and challenged FADD^AEC-KO^ *Ripk3*^−/−^ mice and the respective control mice (*Fadd*^fl/fl^, FADD^AEC-KO^ and *Fadd*^fl/fl^ *Ripk3*^−/−^) with HDM. Comparison of *Fadd*^fl/fl^ *Ripk3*^−/−^ with *Fadd*^fl/fl^ mice revealed that RIPK3 deficiency caused a mildly increased HDM-induced pathology, manifesting with slightly elevated numbers of infiltrating eosinophils and T cells, as well as mildly increased amounts of serum IgE and IL-13, IL-5 and IL-17 protein levels in the lung (Fig. 3A–D). Although this effect was not very strong it was consistent suggesting that systemic RIPK3 deficiency results in a mild exacerbation of HDM-induced airway inflammation. In contrast, RIPK3 deficiency strongly attenuated the exacerbated airway inflammation of FADD^AEC-KO^ mice, with FADD^AEC-KO^ *Ripk3*^−/−^ mice exhibiting HDM-induced pathology similar to *Fadd*^fl/fl^ *Ripk3*^−/−^ mice. Inflammatory cell infiltration in the lungs of FADD^AEC-KO^ *Ripk3*^−/−^ mice was limited in areas around airways and the vasculature similarly to the *Fadd*^fl/fl^ *Ripk3*^−/−^ control mice (Fig. 3A). In line with the histological findings, RIPK3 deficiency attenuated the inflammatory cell recruitment into the lungs since the numbers of eosinophils, T cells, DCs and neutrophils in lungs from double deficient FADD^AEC-KO^ *Ripk3*^−/−^ mice were normalized to the level of the *Fadd*^fl/fl^ *Ripk3*^−/−^ controls (Fig. 3B). Moreover, FADD^AEC-KO^ *Ripk3*^−/−^ mice showed similarly elevated protein levels of cytokines in the lung compared to *Fadd*^fl/fl^ *Ripk3*^−/−^ mice (Fig. 3E). Interestingly, RIPK3 deficiency greatly reduced the expression of *Cxcl13* in the lungs of FADD^AEC-KO^ *Ripk3*^−/−^ mice (Fig. S1B), indicating that RIPK3-dependent necroptosis of FADD deficient AECs induces *Cxcl13* expression and iBALT formation. To investigate whether RIPK3 deletion might have any effect on DC mobilization during the sensitization phase, we assessed the abundance of the various DC subsets in the MLNs 3 days after a single HDM instillation. No significant differences were noted in the numbers of CD11b^+^ and CD103^+^ DCs subsets that migrated to the draining lymph nodes of *Fadd*^fl/fl^ or *Fadd*^fl/fl^*Ripk3*^−/−^ mice, indicating that RIPK3 deficiency did not influence normal DC activation (Fig. 3F). However, RIPK3 deficiency reduced the number of migrated CD103^+^ DCs in the MLNs of FADD^AEC-KO^*Ripk3*^−/−^ mice, suggesting that RIPK3-dependent mobilization of this type of DCs critically contributes to the exacerbated airway inflammatory response to HDM in FADD^AEC-KO^ mice. Taken together, these results showed that RIPK3 deficiency strongly attenuated the exacerbated HDM-induced airway inflammatory phenotype of FADD^AEC-KO^ mice, suggesting that RIPK3-mediated necroptosis is responsible for the exaggerated inflammatory response consistent with the findings on FADD^AEC-KO^ *Ripk1*^D138N/D138N^ mice. However, RIPK3 deficiency also had an effect in mildly increasing HDM-induced airway inflammation in mice with intact FADD signaling, possibly via functions of RIPK3 in cell type(s) other than AECs.

RIPK3 mediates necroptosis by phosphorylating and activating the downstream effector MLKL, however, RIPK3 has also been implicated in necroptosis-independent signaling.^37,38^ To directly assess the role of necroptosis in the exacerbated asthma-like pathology of FADD^AEC-KO^ mice we generated FADD^AEC-KO^*Mlkl*^−/−^ mice (Fig. S3A) and subjected these mice and their littermate controls to the same protocol of sensitization and challenge with HDM. Similar to RIPK3 knockout, MLKL deficiency ameliorated the enhanced inflammatory response in the lungs of FADD^AEC-KO^ mice as judged by histopathological examination (Fig. S3B) and by the attenuated recruitment of immune cells into the lung (Fig. S3C). These results provide additional genetic evidence that activation of RIPK1-RIPK3-MLKL-dependent necroptosis in FADD deficient AECs contributes to the severity of allergic airway inflammation.

**Fig. 4:**
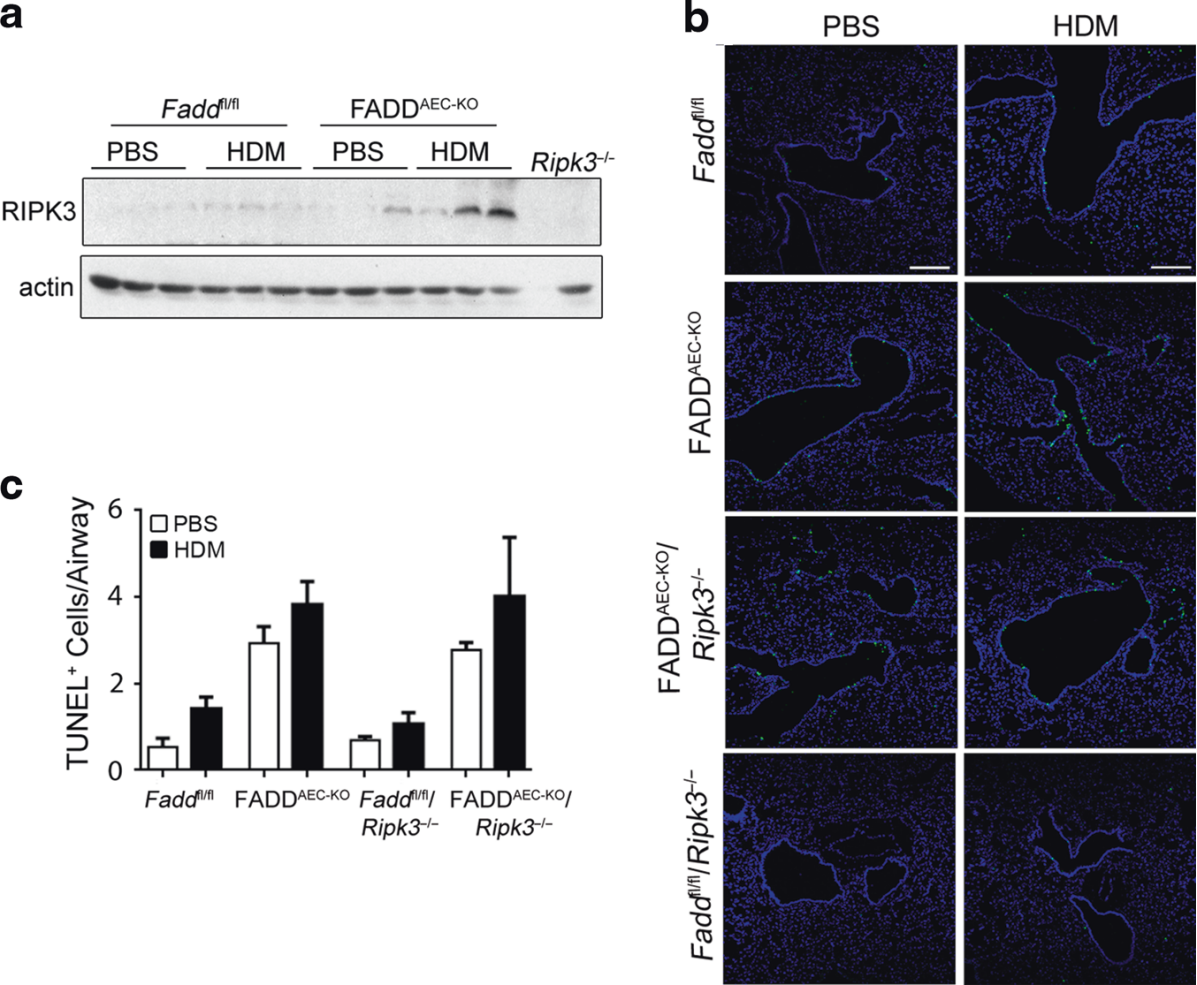
HDM induces death of AECs. **A** Immunoblot for RIPK3 in whole lung lysates 24 h after HDM exposure. **B** Representative images from TUNEL staining in lung sections from mice treated with PBS or HDM for 24 h. Scale bars, 100 μΜ. **C** Quantification of TUNEL^+^ cells from (**B**). Pooled data from two experiments *n* = 3–9 for HDM, *n* = 3–5 for PBS. Error bars indicate SEM. *P* values reflect the Mann–Whitney U test: *P < 0.05, **P < 0.01, ***P < 0.001.

The findings that RIPK1-RIPK3-MLKL signaling is required for the exacerbated HDM-induced lung inflammation in FADD^AEC-KO^ mice suggested that FADD deficiency sensitized AECs to necroptosis after exposure to HDM, which resulted in exaggerated inflammatory responses. We therefore aimed to assess whether FADD-deficiency indeed sensitized AECs to necroptosis. Phosphorylation of MLKL by RIPK3 is an essential step for the induction of necroptosis. We therefore attempted to assess the occurrence of necroptosis in the lungs of mice at different time points after a single intratracheal challenge with HDM by using antibodies against phosphorylated RIPK3 or MLKL. However, unfortunately we could not observe specific staining of RIPK3 or MLKL phosphorylation in lung sections from HDM-treated *Fadd*^fl/fl^ or FADD^AEC-KO^ mice using available antibodies, therefore we could not directly address the occurrence of necroptosis in these tissues (data not shown). Nevertheless, western blot analysis of lung homogenates showed that HDM instillation resulted in increased expression of RIPK3 protein in the lungs of FADD^AEC-KO^ mice compared to WT mice, indicating that RIPK3-dependend necroptosis might take place in these lungs (Fig. 4A).

To circumvent the unsuccessful attempt to detect necroptotic cells by immunostaining with antibodies against phosphorylated RIPK3 and MLKL, we sought to examine the presence of dead cells using terminal deoxynucleotidyl transferase-mediated dUTP nick end labeling (TUNEL) assay, which stains apoptotic but also necrotic cells although it cannot distinguish between these types of cell death.^39^ Administration of HDM but not of PBS caused a transient wave of cell death in WT lungs that started at 5 h (not shown) and peaked at 24 h after instillation (Fig. 4B). TUNEL^+^ cells were mostly evident in the luminal surface of the bronchi and bronchioles and to a lesser extent in the lung parenchyma. HDM caused the appearance of increased numbers of TUNEL^+^ AECs in the lungs of FADD^AEC-KO^ mice (Fig. 4B, HDM and Fig. 4C), consistent with the concept that FADD deficiency may sensitize these cells to necroptosis. However, lung sections from HDM-treated FADD^AEC-KO^*Ripk3*^−/−^ mice showed similar numbers of TUNEL^+^ cells compared to lungs from FADD^AEC-KO^ mice, suggesting that the increased number of TUNEL^+^ cells in FADD^AEC-KO^ mice were not due to RIPK3-mediated necroptosis (Fig. 4B, C). To further investigate this puzzling result, we examined cell death in lungs from PBS-treated mice and found that PBS instillation caused the death of AECs in FADD^AEC-KO^ and FADD^AEC-KO^*Ripk3*^−/−^ mice, to an extent similar to HDM (Fig. 4B, PBS and 4C). In the context of a more broad analysis including also *Scgb1a1*-Cre^Tg^ mice that did not carry any loxP-flanked *Fadd* allele we found that intranasal PBS instillation caused increased AEC death in these animals (Fig. S4A), indicating that the physical/mechanical stress of the instillation procedure causes the death of AECs expressing Cre recombinase. This finding raised the possibility that the exaggerated allergic inflammation in FADD^AEC-KO^ mice might also be related to the unspecific increased cell death caused by expression of Cre recombinase. Therefore, to assess whether the increased AEC death associated with the expression of the *Scgb1a1*-Cre transgene could contribute to the exacerbated HDM-induced airway inflammation in FADD^AEC-KO^ mice, we examined the responses of *Scgb1a1*-Cre^Tg^ mice to HDM sensitization and challenge. *Scgb1a1*-Cre^Tg^ mice developed signs of asthma pathology that were indistinguishable from their *Scgb1a1*-Cre^WT^ littermates, as judged by histopathological analysis (Fig. S4B), BALF inflammatory cell quantitation (Fig. S4C), T_H_2 cytokine secretion by MLN cells (Fig. S4E) and serum IgE production (Fig. S4D). Moreover, qRT-PCR analysis showed that the mRNAs of *Il*-5, *Il-13*, *Il-17* as well as of *Ccl2* were induced equally in the lungs of both *Scgb1a1*-Cre^Tg^ mice and control mice (Fig. S4F). Therefore, the increased occurrence of AEC death due to unspecific Cre effects did not influence the development of HDM-induced asthma pathology. The unspecific death of AECs expressing the *Scgb1a1*-Cre transgene likely masks the presence of necroptotic cells in the lungs of FADD^AEC-KO^ mice precluding the accurate quantification of the extent of necroptosis taking place in these cells. Taken together, these results showed that the exacerbated lung inflammation in HDM-challenged FADD^AEC-KO^ mice is specifically linked to the loss of FADD in AECs and depends on RIPK3, suggesting that increased necroptosis of FADD-deficient AECs contributes to the worsening of HDM-induced asthma.

### AEC-specific FADD deficiency limits HDM-induced mucus hypersecretion and AHR

**Fig. 5:**
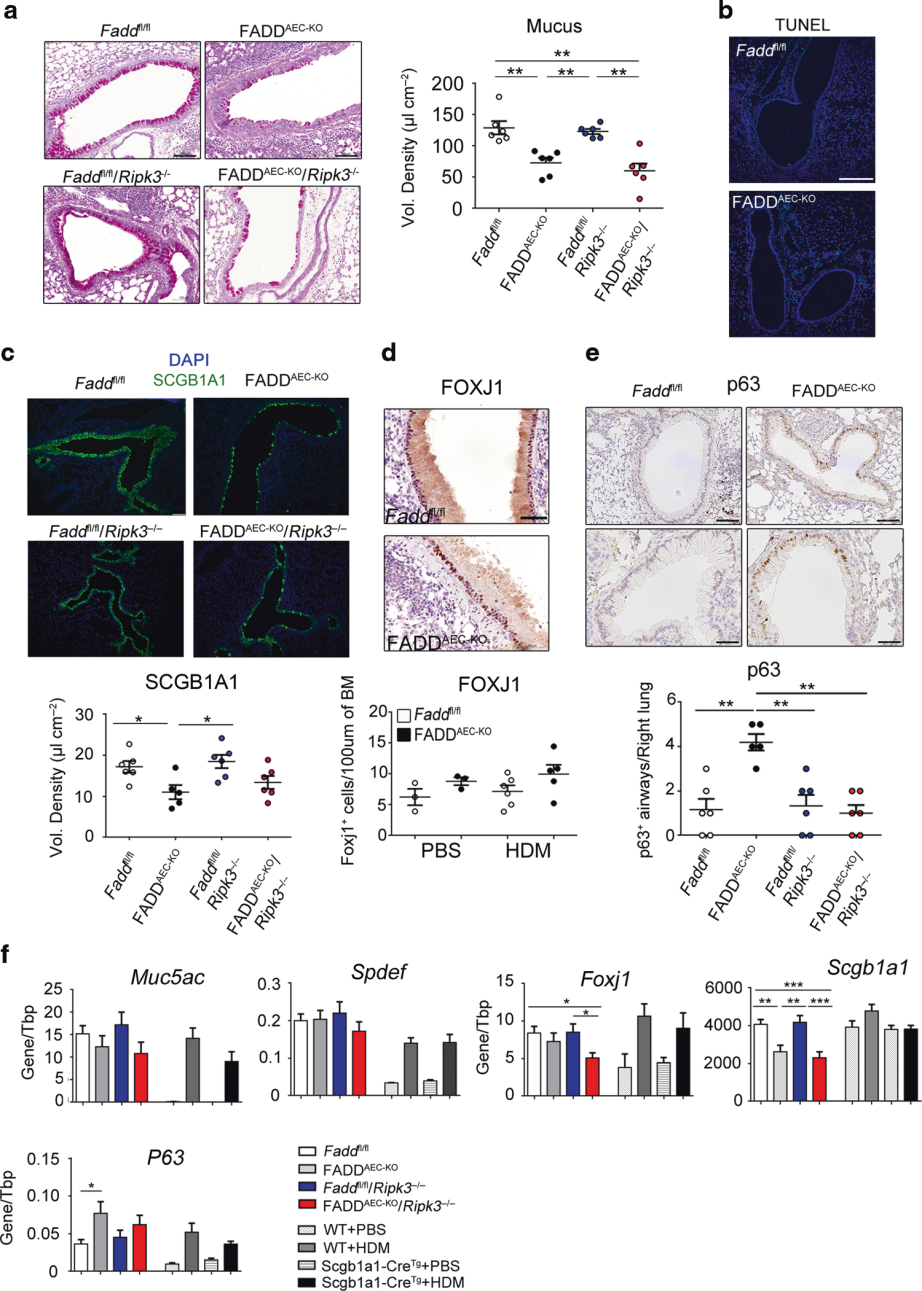
AEC-specific FADD deficiency limits HDM-induced mucus hypersecretion and induces epithelial remodeling. **A** Representative images of PAS stained lung sections and quantification of mucus content from the indicated genotypes after sensitization and challenge with HDM. Scale bar, 100 μΜ. **B** Representative images from lung sections derived from mice after sensitization and challenge with HDM stained with TUNEL. **C**–**E** Representative images and quantification analysis of lung sections derived from mice after sensitization and challenge with PBS or HDM and immunostained for SCGB1A1 (**C**), FOXJ1(**D**) or p63 (**E**). Scale bars for (**C**): 100 μΜ, (**D**, **E**): 50 μΜ. **F** qRT-PCR analysis of the indicated genes in whole lung mRNA isolated from mice with the indicated genotype after sensitization and challenge with HDM. For **A**, **E** *n* = 5–6. For **F** *n* = 9–12, from two experiments. Error bars indicate SEM. *P* values reflect the Mann–Whitney U test: *P < 0.05, **P < 0.01, ***P < 0.001.

Apart from the impact on allergic airway inflammation, FADD deficiency caused additional structural changes to the airways. Histopathologic analysis of PAS-stained lung sections revealed that the airways of FADD^AEC-KO^ mice contained overall less mucus-producing goblet cells when compared to their *Fadd*^fl/fl^ littermates 3 days after the last challenge with HDM (Fig. 5A), despite the increased inflammatory response and the abundance of IL-13. TUNEL staining did not reveal significant ongoing cell death in the airways of either genotype at this stage (Fig. 5B). Although “fragile” AECs are expected to have been washed away during lavage of the lungs, this result indicates that the reduced numbers of goblet cells is not the consequence of increased ongoing death of FADD deficient epithelial cells. The defect in mucus-producing cells prompted us to investigate the fate of other AEC types in the lungs of FADD^AEC-KO^ mice. Staining of lung sections with an antibody against SCGB1A1, a specific marker of Club cells that are the non-ciliated secretory cells of the airways, showed that their numbers were significantly reduced in the airways of HDM-treated FADD^AEC-KO^ mice particularly in severely inflamed bronchi or bronchioles (Fig. 5C). In contrast, the abundance of ciliated cells, identified by immunostaining for the transcription factor FOXJ1, did not differ between FADD^AEC-KO^ and *Fadd*^fl/fl^ littermates (Fig. 5D). Loss of Club cells activates a repair program in which basal cells proliferate to replenish the Club cell compartment through differentiation.^40^ We therefore assessed the presence of basal cells in the airways of FADD^AEC-KO^ and control mice by immunostaining for p63 (Fig. 5E). Indeed, many more airways containing p63^+^ basal cells were detected in the lungs of FADD^AEC-KO^ mice. Interestingly, RIPK3 deficiency limited the number of airways with p63^+^ cells, indicating that RIPK3-dependent mechanisms contribute to the ongoing epithelial cell injury and/or airway remodeling in FADD deficient AECs. Consistent with the immunohistochemistry results, the mRNA levels of *Scgb1a1* were significantly downregulated in the lungs of FADD^AEC-KO^ mice when compared to WT littermates (Fig. 5F). However, expression levels of *Muc5ac*, the major mucin that is induced during allergic airway responses, were mildly reduced. This effect could be explained by the fact that the mRNA expression of *Spdef*, a transcription factor largely responsible for goblet cell metaplasia, was not altered suggesting that the transcriptional program for goblet cell differentiation is induced equally in both FADD^AEC-KO^ and WT mice (Fig. 5F). We hypothesized that Club cell loss due to RIPK3-dependent necroptosis during challenge with HDM might cause a shortage of goblet cell progenitors resulting in less mucus secreting cells. However, RIPK3 deficiency did not restore the abundance of mucus producing cells (Fig. 5A) and only partially restored SCGB1A1 protein expression (Fig. 5C) in FADD^AEC-KO^ mice. Consistently, RIPK3 deficiency did not normalize the expression levels of *Scgb1a1* and *Muc5ac* mRNAs in the lungs of FADD^AEC-KO^ mice (Fig. 5F). These results prompted us to investigate whether the underlying unspecific cell death caused by the expression of Cre recombinase might contribute in parallel with RIPK3-dependent necroptosis to the specific loss of Goblet cells. qRT-PCR analysis showed that the mRNA expression levels of *Muc5ac, Spdef, Foxj1, Scgb1a1 and p63* were not significantly altered in the lungs of *Scgb1a1*-Cre^Tg^ mice compared to WT littermates (Fig. 5F), suggesting that the unspecific cell death associated with Cre expression does not cause the loss of Club cells.

**Fig. 6:**
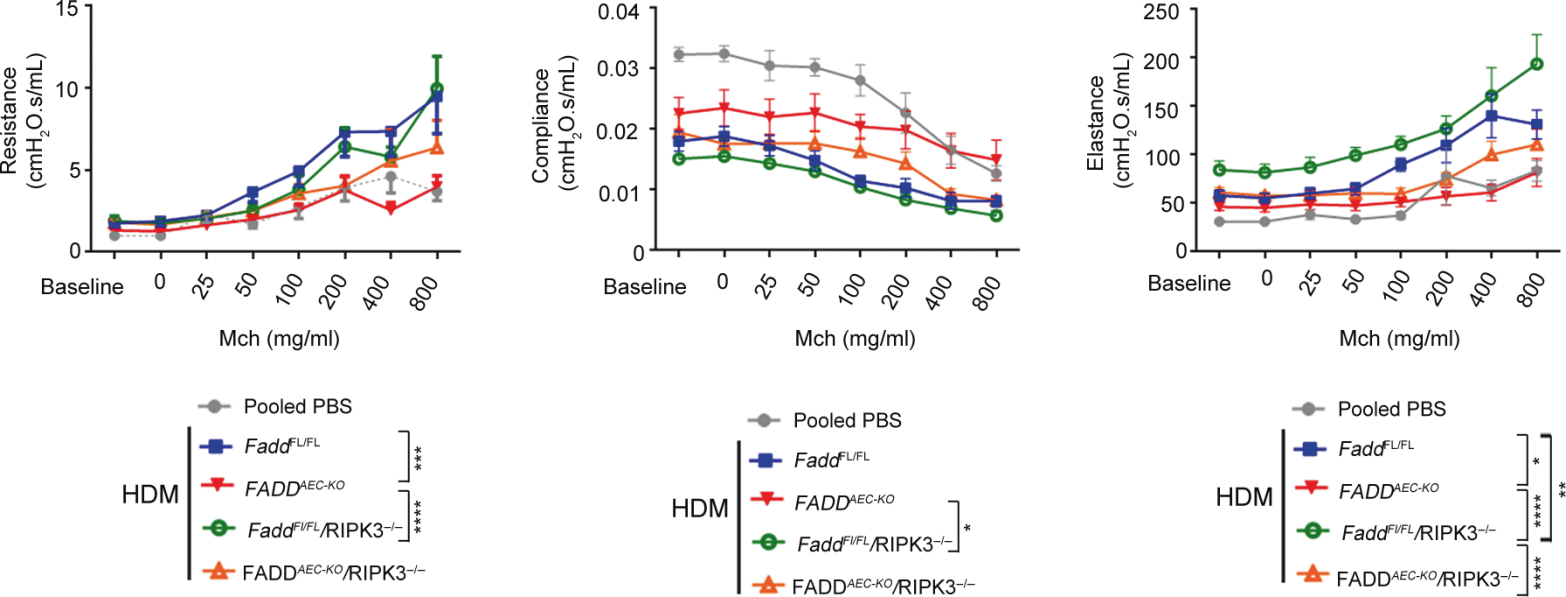
AEC-specific FADD deficiency limits AHR parameters. Resistance, compliance and elastance measurements of lung function in response to increasing doses of methacholine, as assessed with the flexivent (SCIREQ). *n* = 7 for PBS (pooled data), *n* = 6 for HDM from one experiment. Error bars indicate SEM, *p* values reflect 2 way ANOVA test: *p < 0.05, **p < 0.001, ***p < 0.001. *p* values for the 800 mg/ml methacholine dose are depicted.

In asthma, the degree of inflammation is thought to correlate with airflow obstruction and AHR development due to the ensuing activation of smooth muscle cells. A recent study, using *Muc5ac*^−/−^ mice showed that even in the presence of ongoing inflammation lack of mucus hypersecretion abolishes AHR.^41^ Given the phenotype of FADD^AEC-KO^ mice, in which an exacerbated inflammatory response coincided with less mucus production, we were interested to determine the net contribution of these clinical entities to AHR development. As expected, lung resistance in HDM-treated *Fadd*^fl/fl^ mice increased considerably upon methacholine exposure (Fig. 6). Elastance values were also increased and conversely compliance was reduced. *Ripk3*^−/−^ mice developed AHR similar to *Fadd*^fl/fl^ mice. Surprisingly, AEC-specific deletion of FADD resulted in significant protection from AHR since resistance, compliance and elastance values were similar to those of PBS-treated mice. HDM administration in *Ripk3*^−/−^ mice caused worsening of lung function in a manner similar to *Fadd*^fl/fl^ mice. Interestingly, RIPK3 expression mediated at least to some extent the observed amelioration of AHR in FADD^AEC-KO^ mice, since RIPK3 deficiency partly increased the resistance and elastance parameters in FADD^AEC-KO^*Ripk3*^−/−^ mice, suggesting that RIPK3-dependent mechanisms contribute to the amelioration of lung function in FADD^AEC-KO^ mice. These results support the concept that mucus hypersecretion contributes to airway obstruction and AHR development despite the ongoing inflammation.

## Discussion

Regulation of epithelial cell survival and death has emerged as a key mechanism affecting immune homeostasis in barrier tissues such as the skin and the intestine. Previous studies showed that conditional targeting of FADD or Caspase 8 caused epithelial cell necroptosis resulting in severe inflammation in both the skin and the intestine.^24–26,42,43^ In addition, epithelial cell specific RIPK1 deficiency caused skin inflammation by sensitizing keratinocytes to ZBP1-RIPK3-MLKL-mediated necroptosis,^31,34,44,45^ and severe intestinal pathology by sensitizing intestinal epithelial cells to FADD-caspase-8-mediated apoptosis.^31,32^ The lung epithelium also constitutes a barrier against inhaled pollutants, allergens and pathogens, therefore we wanted to address whether regulation of epithelial cell death also controls tissue homeostasis in the lung. Our results presented here show that AEC-specific ablation of FADD or RIPK1 did not cause spontaneous death of epithelial cells and did not disturb normal tissue homeostasis in the lung, in contrast to the findings in the skin and the intestine. While it remains unclear why the lung epithelium responds differently from the skin and intestinal epithelium to FADD or RIPK1 deficiencies, this is likely related to the relatively lower microbial load in the airways compared to the surface of the skin and the lumen of the gut.

Whereas FADD ablation in AECs did not disturb normal lung homeostasis under steady-state conditions, it caused exacerbated asthma-like lung pathology in response to HDM sensitization and challenge. AEC-specific FADD deficiency did not affect the qualitative features of the immune response against HDM, but rather amplified the underlying type 2 immune response. Our experiments showed that inhibition of RIPK1 kinase activity, by crossing to mice expressing kinase-inactive RIPK1, as well as RIPK3 or MLKL deficiency greatly attenuated the severity of airway inflammation in FADD^AEC-KO^ mice, suggesting that RIPK1-RIPK3-MLKL-mediated necroptosis of FADD-deficient AECs strongly contributes to the exacerbation of HDM-induced asthma. Lack of RIPK1 kinase activity or RIPK3 or MLKL deficiency did not protect mice with intact FADD signaling from HDM-induced airway inflammation, showing that RIPK1-RIPK3-MLKL-mediated necroptosis does not play an important role in HDM-induced asthma in wild type mice. The exact mechanisms by which necroptosis of FADD-deficient AECs potentiates type 2 inflammation are not yet fully understood. However, increased numbers of CD103^+^ DCs, which are known to present antigens in association with dead cells,^46^ were found to migrate in MLNs of FADD^AEC-KO^ but not of FADD^AEC-KO^ *Ripk3*^−/−^ mice after HDM instillation, suggesting that AEC necroptosis contributes to the increased activation and expansion of this type of DCs. FADD deficiency resulted in a mild amplification of the underlying T_H_2 immune response against HDM, which was dependent on RIPK1-RIPK3 signaling as both the inhibition of RIPK1 kinase activity and RIPK3 deficiency limited the excessive production of type 2 cytokines in the lymph nodes and reduced the expression of type 2 cytokines in lung tissue from FADD^AEC-KO^ mice. Interestingly, lack of RIPK1 kinase activity, and to a lesser extent RIPK3 deficiency, limited the activation of T_H_17 cells, which is associated with more severe asthma endotypes,^2^ in FADD^AEC-KO^ mice, suggesting that in the context of type 2 immunity in the lung, AEC necroptosis might potentiate neutrophil-associated T_H_17 pathogenic responses. These findings are consistent with the role of necroptosis in driving inflammatory responses upon epithelial-specific ablation of FADD or caspase-8 in the intestine and the skin,^24,25,43^ and identify necroptosis as a potent amplifier of allergic lung inflammation. HDM could mediate necroptosis of FADD-deficient AECs directly by activating TLR4 and/or indirectly by inducing the expression of death receptor ligands such as TNF and TRAIL that are known to be produced during allergic responses.^47^

While our genetic studies support a role of necroptosis in driving the exacerbated HDM-induced lung pathology in FADD^AEC-KO^ mice, we were not able to provide definite evidence of the presence of necroptotic cells in the lungs of HDM-challenged animals due to the unavailability of antibodies capable of reliably and specifically detecting necroptotic cells in situ in tissue sections. Nevertheless, HDM instillation caused an upregulation of RIPK3 expression in the lungs of FADD^AEC-KO^ mice, which could be indicative of ongoing necroptotic cell death of FADD deficient AECs. Our attempt to detect and quantify dying cells using TUNEL staining was also hindered by the finding that expression of the *Scgb1a1-Cre* transgene in the absence of loxP-flanked alleles sensitized AECs to death in response to instillation of HDM but also PBS. Whereas this unspecific cell death associated to Cre recombinase expression prevented the accurate assessment of necroptosis caused by FADD deficiency, the finding that *Scgb1a1-*Cre^Tg^ mice did not develop more severe allergic inflammation to HDM demonstrated that the exacerbated responses in FADD^AEC-KO^ mice were due to FADD deficiency and not the expression of Cre. These results also showed that the *Scgb1a1*-Cre^Tg^ mice can be used for the study of allergic inflammation in AEC-specific conditional knockout mice, however any assessment of cell death in these mutants needs to be rigorously controlled taking into account the unspecific cell death associated with the Cre transgene.

Despite the exacerbated inflammatory response, FADD^AEC-KO^ mice were protected from AHR development. This protection was associated with reduced numbers of mucus-producing cells in the airways of these mice, indicating that airway obstruction due to mucus hypersecretion contributes substantially to AHR in asthma. The most plausible scenario for the reduced numbers of Goblet cells in the airways of FADD^AEC-KO^ mice is the death of their precursors i.e., the Club cells, as it was also evident by our analysis. However, RIPK3-dependent necroptosis cannot fully account for the reduced number of Goblet cells since RIPK3 deficiency did not restore their numbers. An alternative explanation is that cell death due to Cre expression contributes in parallel with necroptosis to the demise of Club cells. Collectively and taking into account the aforementioned complications, these results support recent findings suggesting that either genetic^41^ or pharmacologic^48^ targeting of mucus or mucus producing cells could be a beneficial strategy for the control of AHR during asthma.

Necroptosis is considered as a fail-safe cell death mode that operates in certain conditions such as for example when apoptosis is inhibited. This is best illustrated in the case of viral infections. In their attempt to evade host defences and to propagate within cells, viruses have evolved mechanisms that interfere with cell death induction. For example, viruses are known to express caspase inhibitors that are able to block apoptosis execution.^49^ Some viruses in addition to inhibition of apoptosis express proteins that interfere with RIPK1/RIPK3 signaling leading to inhibition of necroptosis, facilitating their replication in the infected cells.^49^ Due to its lytic nature, necroptosis is considered to be an immunogenic form of cell death as opposed to apoptosis that is thought to elicit no or weak immune responses. Therefore, whereas necroptosis is considered an important anti-viral defense mechanism, its beneficial effects in preventing viral replication and spread are often outweighed by its propensity to trigger exagerated immune responses that ultimately can cause tissue damage. For example Influenza virus infection induces necroptosis-depended hyperinflammation that contributes to the severity of the disease.^50^ Intriguingly, the extent to which FADD deficiency aggravated type 2 inflammation is reminiscent of the reported amplification of allergic airway inflammation induced by respiratory viral infections.^51,52^ Respiratory viruses such as Rhinovirus (RV) and Respiratory Syncytial Virus (RSV) have severe effects for patients at risk or with existing asthma.^53^ However, the mechanisms by which infections in the respiratory tract lead to exacerbation of the underlying allergic inflammation are poorly understood.^53^ It was shown recently that the RV 3C protease can modulate cell death by cleaving RIPK1 resulting in suppression of apoptosis and induction of caspase-independent cell death for release of viral particles,^54,55^ indicating that viral infection of AECs in the lungs can interfere with RIPK1/RIPK3 mediated cell death pathways. In a more recent study, RSV infection was shown to induce necroptotic death of human AECs and that both genetic and pharmacologic inhibition of necroptosis during viral infection in neonatal mice decreased acute bronchiolitis and protected mice from the development of asthma-like pathology upon exposure to cockroach allergen later in life.^56^ In addition, it has been reported that transgenic mice overexpressing human gasdermin B (GSDMB), which induces lytic cell death resembling pyroptosis, developed asthma-like pathology.^57^ GSDMB is located at the 17q21 locus that is consistently associated with asthma and a splice variant that abolishes the ability to execute pyroptosis in bronchial epithelial cells was recently associated with lower asthma risk.^58^ Collectively, our results in FADD^AEC-KO^ mice combined with the above observations suggest a possible causative relationship between viral-induced cell death of AECs and exacerbation of allergic airway pathology. These findings warrant further studies to address whether necroptosis inhibition, e.g., using RIPK1 kinase inhibitors,^59^ could prevent asthma exacerbation by viral infections.

## Materials & methods

### Mice

*Fadd*^fl/fl^,^25^*Ripk3*^*−/−*^,^60^*Ripk1*^fl/fl^,^31^*Ripk1*^D138N/D138N^^36^ and *Scgb1a1*-cre mice (CBA/C57BL6 F1)^29^ were described previously. *Scgb1a1*-cre mice were backcrossed for 2–3 generations with C57Bl6 mice before they were used for the generation of conditional knock-out mice. For the generation of *Mlkl*^−/−^ mice, Cas9 mRNA together with the sgRNA targeting the *Mlkl* gene (5′-CGTCTAGGAAACCGTGTGCA-3′) were microinjected into the pronucleus of fertilized oocytes obtained from *Fadd*^*fl/fl*l^ mice. On the next day, the injected embryos were transferred to foster mothers and allowed to develop to term. An *Mlkl* allele shown to have a 35 bp deletion at exon 2 resulting in a premature stop codon was propagated as the *Mlkl* knockout allele used for this study (Fig. S3A). Mice were maintained at the SPF animal facilities of the Institute for Genetics and the CECAD Research Center of the University of Cologne, under a 12 h light cycle, and given a regular chow diet (Harlan, diet number 2918 or Prolab Isopro RMH3000 5P76) ad libitum. All animal procedures were conducted in accordance with European, national and institutional guidelines and protocols were approved by local government authorities (Landesamt für Natur, Umwelt und Verbraucherschutz Nordrhein-Westfalen, Germany). Mice of the indicated genotype were assigned at random to groups.

### House dust mite-induced asthma model

On day 0, mice were lightly anesthetized with ketamine (80 mg/Kg Ketamine, 8 mg/Kg xylazine) and received 1 µg HDM (Greer laboratories) intranasally dissolved in 50 μl of PBS. On days 7–11, mice were challenged daily with 10 µg HDM intranasally. On day 14, mice were euthanized. For the acute model of HDM mice were instilled intranasally with either PBS or HDM (100 μg in 50 μl) and sacrificed 24 h later. All related assays for the analysis of the immune response have been reported previously^15,30,31,61^ and are presented in detail in an online supplement.

### Statistical analyses

Data shown in column graphs represent the mean ± s.e.m. For all experiments, we calculated the difference between groups with the Mann–Whitney U test for unpaired data (GraphPad Prism). Differences were considered significant when *p* value was lower than 0.05. *P ≤ 0.05, **P ≤ 0.01, ***P ≤ 0.005.

## Supplementary Information

Supplementary material
