# Supplementary material for: Airway epithelial cell necroptosis contributes to asthma exacerbation in a mouse model of house dust mite-induced allergic inflammation
Source: Mucosal Immunol. 2021 May 27;14(5):1160–71. doi: 10.1038/s41385-021-00415-5 (PMC8379077; doi:10.1038/s41385-021-00415-5)
Supplement: Supplementary file 1 — Supplementary material [file 41385_2021_415_MOESM1_ESM.pdf]

Airway epithelial cell necroptosis contributes to asthma exacerbation in a mouse model of house dust mite-induced allergic inflammation

Nikos Oikonomou, Martijn J Schuijs, Antonios Chatzigiagkos, Ariadne Androulidaki, Vassillis Aidinis, Hamida Hammad, Bart N Lambrecht, Manolis Pasparakis

Online data supplement

Supplementary material

Material and methods

House dust mite-induced asthma model

On day 0, mice were lightly anesthetized with ketamine (80 mg/Kg Ketamine, 8 mg/Kg xylazine) and received 1μg HDM (Greer laboratories, Lenoir, NC, USA) intranasally dissolved in 50 μl of PBS. On days 7-11, mice were challenged daily with 10 μg HDM intranasally. On day 14, mice were euthanized. Blood was obtained from the iliac vein and serum was prepared. Bronchoalveolar lavage (BAL) was performed using 3x1ml of EDTA-containing PBS. Cells were collected after centrifugation at 1300 rpm for 10 min at 4^0C and the cleared BAL fluid was frozen at -80^0C. The left lung was snap frozen for RNA extraction. The right lung was instilled with 4% PFA, excised and incubated overnight in 4% PFA before paraffin embedding. Single cell suspensions were obtained from mediastinal lymph nodes by homogenization through a 100 μm cell sieve. Cells were restimulated *ex vivo* with 15 μg/ml HDM for 3 days, supernatants were collected and the levels of IL-13, IL-5 and IL-17 were measured with ELISA kits from eBiocience, following the manufacturer’s protocol. Lung function was performed using an invasive measurement of dynamic resistance (Flexivent, Scireq, Montreal, CA).

Serum IgE levels

Total IgE levels in serum were measured with an ELISA using Purified Rat Anti-Mouse IgE (Clone R35-92, BD, Franklin Lakes, NJ, USA) as a capture antibody and Biotin Rat Anti-Mouse IgE (Clone R35-72, BD) as the detection antibody. Color development was achieved by incubation with TMB (3,3′,5,5′-Tetramethylbenzidine) Substrate (Sigma, St Louis, MI, USA), following the addition of Streptavidin-HRP Ab (#18-152, Millipore, Burlington, MA, USA). Color development was stopped with the addition of a 2N H_2SO_4 solution.

Acute effects of HDM instillation

Mice were instilled intranasally with either PBS or HDM (100 μg in 50 μl) and sacrificed 24 h later. After perfusion of the lungs the left lung was snap frozen for qRT-PCR analysis or protein quantification. The right lung was instilled with 4% PFA, excised and incubated overnight in 4% PFA before paraffin embedding.

Isolation of Club cells

Club cells were isolated with a previously described method [1]. Briefly, lungs were instilled with 0,25% trypsin/HBSS and after dissection they were incubated in DMEM at 37°C for 20 min. After neutralization of trypsin with FBS, a single cell lung suspension was obtained after mincing the digested tissue and filtering through 40 μm strainers. The cells were incubated at 37°C for 2 h to remove adherent populations and Club cells in the supernatant were isolated by centrifugation at 30g for 8 min.

Immunoblotting

Lung tissue was homogenized in 1 ml of lysis buffer (200 mM NaCl, 1% NP-40, 10 mM Tris-HCl pH 7.5, 5 mM EDTA and 2 mM DTT) supplemented with protease and phosphatase inhibitors (Roche, Basel, CH) using a Precyllis 24 homogenizer (Bertin, Montigny-le-Bretonneux, FR). Club cells were also lysed in lysis buffer. Tissue or cell lysates were separated on SDS–PAGE and transferred to PVDF membranes (IPVH00010, Millipore). Membranes were probed with primary antibody against RIPK3 (Enzo, Farmingdale, NY, USA), FADD (Sigma), Cre (Sigma) and β-actin (Santa Cruz Biotechnology, Dallas, TX, USA) followed by secondary HRP-coupled antibodies (GE Healthcare and Jackson ImmuneResearch, Westgrove, PA, USA) and developed with chemiluminescent detection substrate (GE Healthcare, Chicago, IL, USA and Thermo Scientific, Waltham, MA, USA).

FACS Analysis

To analyze migratory DCs in the mediastinal lymph nodes, mice were instilled intranasally with either PBS or HDM (100 μg in 50 μl) and sacrificed 3 days later. MLNs were isolated and after dissociation they were digested in a solution containing 0.15 U/ml Liberase TM (Roche) and 10 μg/ml DNaseI (Sigma) at 37^{0}C for 30 min. Single cells were isolated through a 70 μM strainer, counted and used for staining as described previously [2]. The following antibodies were used: CD3 (145-2C11), CD19 (eBio1D3), CD11c (3.9), MHCII (M5/114.15.2), CD64 (X54-5/7.1), CD103 (M290), CD11b (M1/70) and FcεRI (Mar-01) (all from eBioscience/Thermo Scientific). Dead cells were excluded from the analysis by staining with Fixable Viability Dye (eBioscience). Cells were acquired on an LSRFortessa cytometer (BD). Analysis was performed using the Flowjo software.

For analysis of the cellular composition of BALF, cells were stained with CD3, CD19, CD11c, MHCII, CD11b, SiglecF (E50-2440, eBioscience) and Gr-1 (RB6-8C5, eBioscience) antibodies.

Dead cells were excluded from the analysis by staining with Fixable Viability Dye (eBioscience). Cells were analyzed in a LSRFortessa cytometer.

qRT-PCR

Total RNA from lung tissue was extracted with Trizol Reagent (Life Technologies) and RNeasy Columns (Qiagen, Hilden, DE) and cDNA was prepared with Superscript III cDNA-synthesis Kit (Life Technologies). qRT–PCR was performed with TaqMan probes (Life Technologies/Thermo Scientific). TATA-box-binding protein (Tbp) was used as a reference gene. Data were analyzed according to the ΔCt method.

Histology and Immunohistochemistry

Lung tissues were fixed in 4% paraformaldehyde, embedded in paraffin, and cut at 4 µm sections. For histopathological analysis, hematoxylin and eosin staining and periodic acid Schiff (PAS) staining was performed according to standard protocols.

For the quantification of allergic inflammation in the lungs of mice after sensitization and challenge, hematoxylin and eosin stained sections were scored blindly using the semi-quantitative scoring system in Table 1[3].

For immunohistochemical (IHC) staining paraffin sections were rehydrated and heat-induced antigen retrieval was performed in citrate buffer at pH 6.0. Primary antibodies for IHC were goat anti-CC10 (T-18) and goat anti-FADD (M-19) from Santa Cruz biotechnology. Mouse anti-Foxj1 was from eBioscience and mouse anti-p63 was from DAKO. For IHC staining, sections were visualized with the ABC Kit Vectastain Elite (Vector Laboratories, Burlingame, CA, USA) or Streptavidin-HRP (Millipore) and DAB substrate (DAKO/Agilent, Santa Clara, CA, USA and Vector Laboratories). Incubation times with DAB substrate were equal for all samples. For immunofluorescence anti-goat biotinylated antibody was from Jackson Immunoresearch, anti-mouse biotinylated was from Biozol (Eching, DE) whereas Streptavidin-Alexa 488 or Alexa 594 were from Molecular Probes (Thermo Scientific). For the *in situ* detection of dead cells the DeadEnd Fluorometric TUNEL System from Promega (Madison, WI, USA) was used.

For quantification of dead cells, all $TUNEL^+$ cells in affected airways, in four of the five lobes of the lungs were counted. The total count of $TUNEL^+$ cells was divided by the number of airways (bronchi and bronchioles) that were included in the analysis and is expressed as $TUNEL^+$ cells per airway. At least 50 airways per right lung were included in the analysis.

For quantification of airway mucus content, sections of lung tissue were stained with PAS and counterstained with haematoxylin. Mucus content was assessed with ImageJ. Captured images (3 images/lobe) were de-convoluted, thresholded and the airway PAS^{+} stained area and the length of the basement membrane were calculated. Mucus content of the airways is expressed as volume densities (volume per unit surface area of basement membrane) [4]. For Scgb1a1 quantification, images were processed with ImageJ and both area and length of basic membrane were calculated. Scgb1a1 content is expressed as volume density. For Foxj1 quantification, DAB-stained images were de-convoluted, thresholded and $Foxj1^{+}$ nuclei were counted in 4 images/lung. Results are expressed as $Foxj1^{+}$ nuclei/100 um of basic membrane length. For p63, airways containing at least one $p63^{+}$ cell in all four lobes of the right lung were counted.

Table 1. Semi-quantitative scoring system for the evaluation of peribronchial and parenchymal inflammation

Score	Peribronchial inflammation	Parenchymal inflammation
0	No infiltration	No infiltration-no alveolar damage
1	mild (light perivascular/peribronchiolar involvement)	foci with scattered intraalveolar immune cells –normal alveolar wall structure
2	moderate (many vessels and airways affected by substantial numbers of inflammatory cells)	Foci with substantial number of intraalveolar immune cells thickening of the alveolar wall proteinaceous debris
3	severe (generalized accumulations of perivascular/peribronchiolar inflammatory cells with frequent circumferential and/or bridging infiltrates)	Extensive accumulation of inflammatory cells within alveolar spaces involving at least one lobe of the lung

References

1. Maelfait J, Roose K, Vereecke L, Mc Guire C, Sze M, Schuijs MJ *et al.* A20 Deficiency in Lung Epithelial Cells Protects against Influenza A Virus Infection. *PLOS Pathogens* 2016; **12**(1)**:** e1005410.

2. Plantinga M, Guilliams M, Vanheerswynghels M, Deswarte K, Branco-Madeira F, Toussaint W *et al.* Conventional and Monocyte-Derived CD11b+ Dendritic Cells Initiate and Maintain T Helper 2 Cell-Mediated Immunity to House Dust Mite Allergen. *Immunity* 2013; (0).

3. Lafkas D, Shelton A, Chiu C, de Leon Boenig G, Chen Y, Stawicki SS *et al.* Therapeutic antibodies reveal Notch control of transdifferentiation in the adult lung. *Nature* 2015; **528:** 127.

4. Piccotti L, Dickey BF, Evans CM. Assessment of Intracellular Mucin Content In Vivo. *Methods in molecular biology (Clifton, NJ)* 2012; **842:** 279-295.

Supplemental Figures

Figure S1

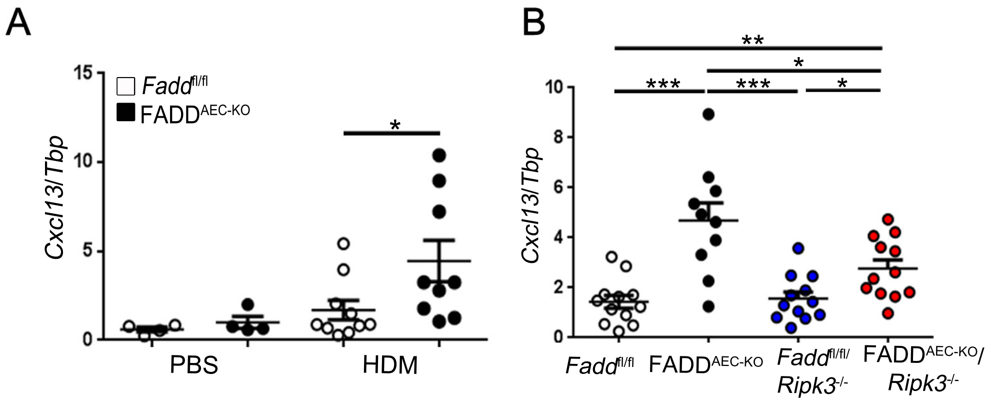

Figure S1. Increased *Cxcl13* expression in FADD^{AEC-KO} mice depends on RIPK3 signaling. (A and B) qRT-PCR analysis of mRNA from mice with the designated genotype, 3 days after the last challenge with HDM. For **(A)** n=4 for PBS, n=9-10 for HDM. For **(B)** n=11-12 for HDM. Pooled data from two experiments. Error bars indicate SEM. P values reflect the Mann-Whitney U test: $^*P < 0.05$, $^{**}P < 0.01$, $^{***}P < 0.001$.

Figure S2

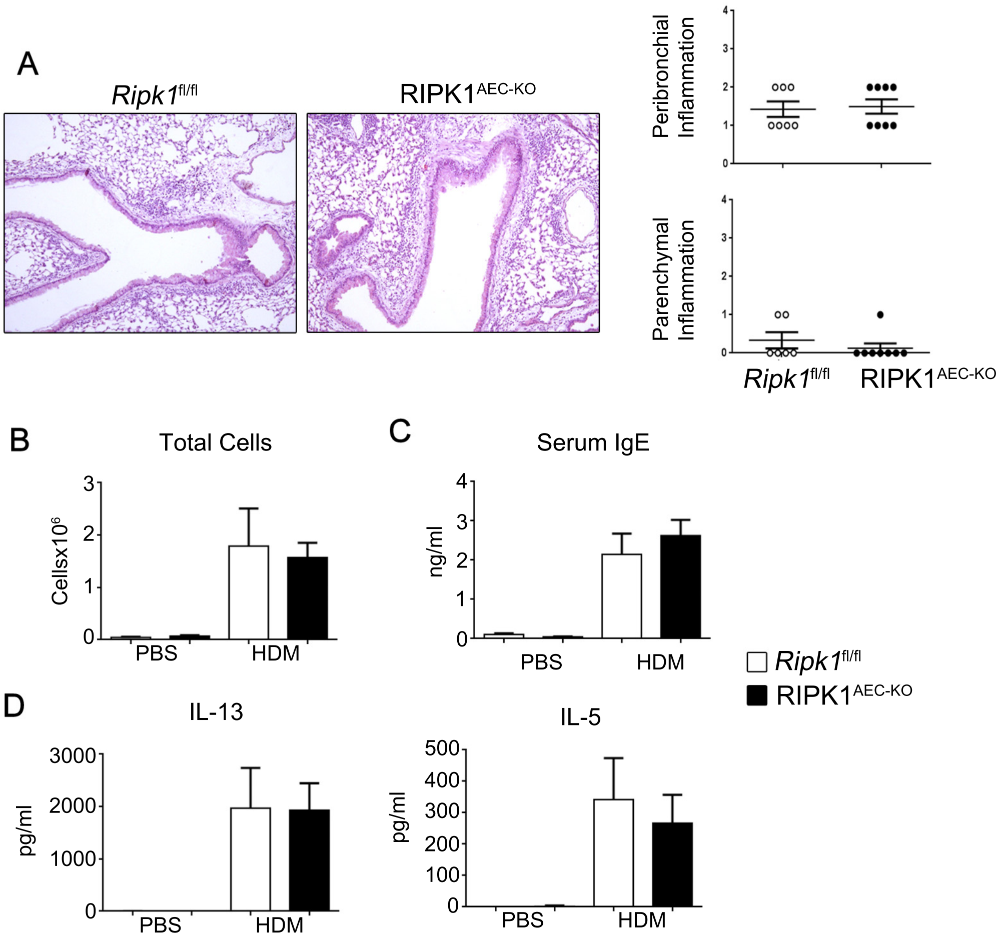

Figure S2. AEC-specific RIPK1 expression is dispensable for HDM-induced allergic inflammation. (**A**) Representative images of H&E stained lung sections from the indicated genotypes sensitized and challenged with PBS or HDM. Scale bar 100μM. (**B**) Total cellularity in BALF. (**C**) Serum levels of total IgE. (**D**) Levels of cytokines in the supernatants of MLN cells after *ex vivo* stimulation with HDM. Pooled data from two experiments, n=7-8 for HDM, n=3-4 for PBS. Error bars indicate SEM. P values reflect the Mann-Whitney U test.

Figure S3

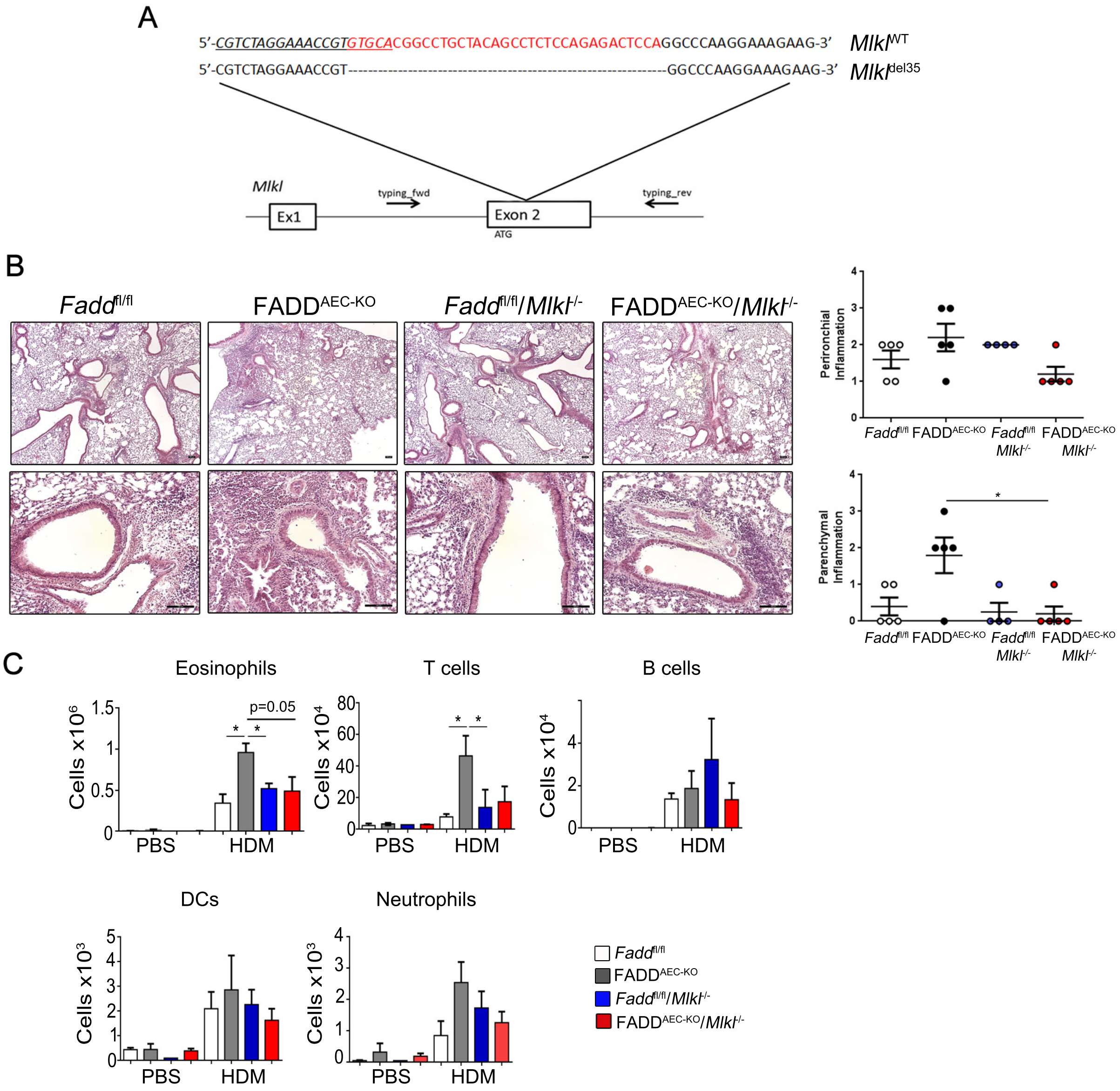

Figure S3. MLKL-dependent AEC necroptosis causes exacerbated HDM-induced airway inflammation in FADD$^{AEC\text{-}KO}$ mice. (A) Sequence of *Mlkl* exon 2 that was targeted by the sgRNA (underlined) and the resulting 35bp deleted allele (in red). **(B)** Representative images of H&E stained lung sections from the indicated genotypes sensitized and challenged with PBS or HDM and clinical scores of peribronchial and parenchymal inflammation. Scale bar 100μM. **(C)** Differential cell count analysis with flow cytometry in BALF cells from the indicated genotypes. n=4-5 from one experiment. Error bars indicate SEM. P values reflect the Mann-Whitney U test: *P < 0.05, **P < 0.01, ***P < 0.001

Figure S4

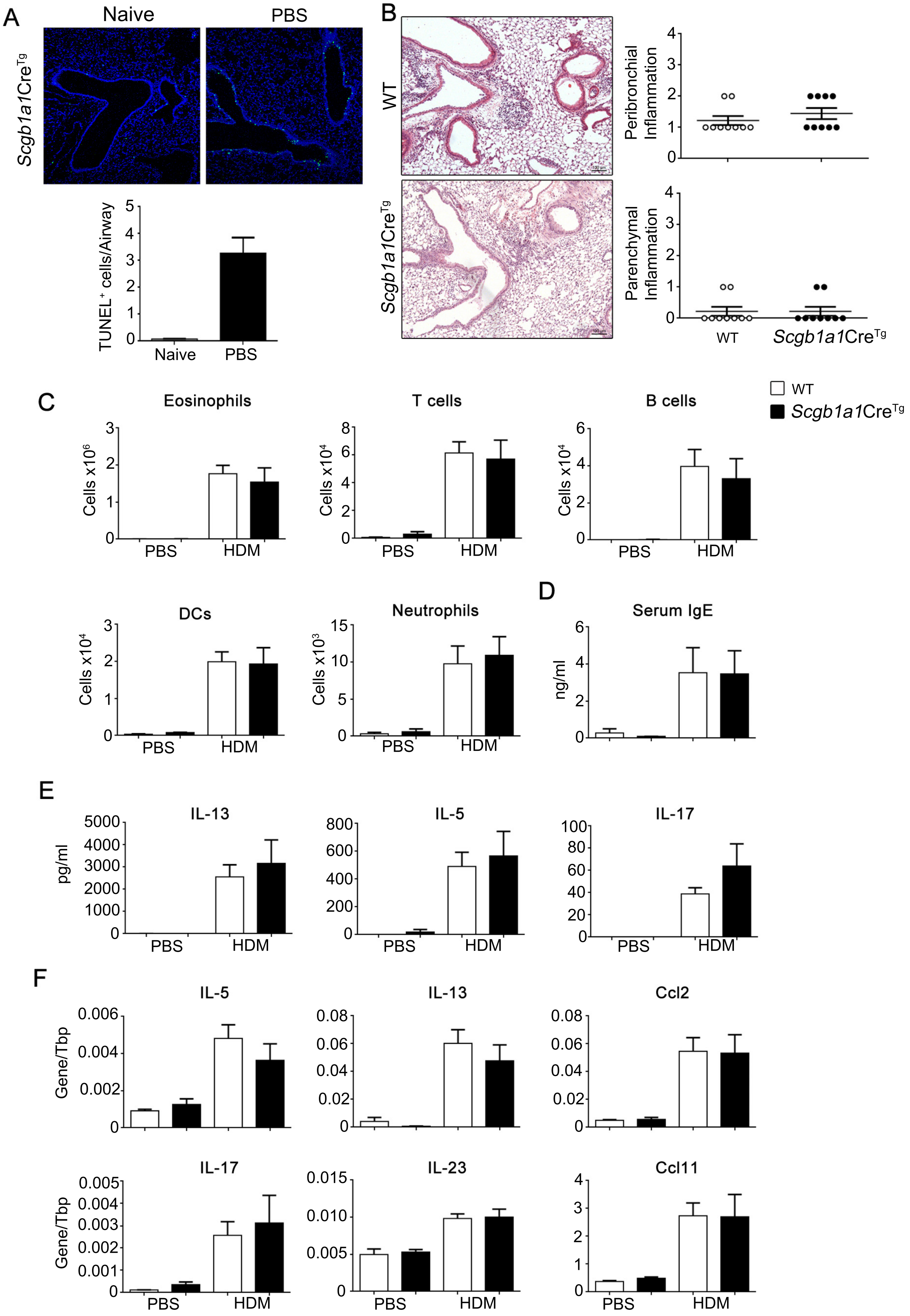

A
Naive
PBS
Scgb1a1CreTg
TUNEL+ cells/Airway
Naive
PBS
B
WT
Scgb1a1CreTg
Peribronchial Inflammation
Parenchymal Inflammation
WT
Scgb1a1CreTg
C
WT
Scgb1a1CreTg
Eosinophils
T cells
B cells
Cells x10^6
Cells x10^4
PBS
HDM
DCs
Neutrophils
Cells x10^3
D
Serum IgE
ng/ml
E
IL-13
IL-5
IL-17
pg/ml
F
IL-5
IL-13
Ccl2
IL-17
IL-23
Ccl11
Gene/Tbp

Figure S4. Development of HDM-induced immunopathology is not affected by unspecific Cre effects. (**A**) Representative images from TUNEL staining and quantification in lung sections from naïve mice or from treated mice 24h after intranasal instillation of PBS. (**B**) Representative images of H&E stained lung sections from WT and *Scgb1a1*-Cre^{Tg} mice sensitized and challenged with PBS or HDM and clinical scores of peribronchial and parenchymal inflammation. Scale bar 100μM. (**C**) Differential cell count analysis with flow cytometry in BALF cells from the indicated genotypes. (**D**) Serum levels of total IgE. (**E**) Levels of cytokines in the supernatants of MLN cells after *ex vivo* stimulation with HDM. (**F**) qRT-PCR analysis of the indicated cytokines in whole lung mRNA. Pooled data from two experiments, n=8-10 for HDM, n=3-4 for PBS. Error bars indicate SEM. P values reflect the Mann-Whitney U test: $^{*}P < 0.05$, $^{**}P < 0.01$, $^{***}P < 0.001$
